# Alopecia Areata and Demyelination as Paraneoplastic Manifestation in Paediatric Hodgkin's Lymphoma

**Published:** 2018-04-01

**Authors:** Shravan Kanaparthi, Shrikiran Aroor, Suneel C Mundkur, Sowmya Shashidhara, Kasi Viswanath Reddy

**Affiliations:** Department of Paediatrics, Kasturba Medical College, Manipal University, Manipal, Karnataka, India

**Keywords:** Hodgkin's lymphoma, Paraneoplastic syndrome, Alopecia areata, Pontine myelinolysis

## Abstract

Hodgkin’s Lymphoma is one of the commonly encountered lymphomas in childhood. Most of the children present with lymphadenopathy. A rare subset of children do present with constellation of atypical symptoms as paraneoplastic syndromes. We hereby present an 11-year-old boy with classical Hodgkin’s Lymphoma associated with Alopecia areata and demyelination as paraneoplastic manifestations. Both these paraneoplastic manifestations improved after initiating chemotherapy (ABVD regimen). A high index of suspicion for underlying malignancy would help clinicians in clinching an early diagnosis and would avert the associated complications.

## Introduction

 Hodgkin’s lymphoma, a lymphoreticular malignancy, was first described in 1832 by Dr Thomas Hodgkin^1^^.^ It accounts for 5-8% of childhood cancers with different age distribution among developed and developing countries^2^^,^^3^. It has an incidence of 2.4/100000 person-years with the classical type of Hodgkin’s lymphoma being the most common. With the currently available treatment, it is one of the most curable childhood malignancies. It has a bimodal age distribution with an initial peak around 20-30yrs and a second peak in adults over 55years of age.^4^ Though lymphadenopathy is one of the most common manifestations, atypical presentations initially in the form of paraneoplastic syndromes can occur, often delaying the diagnosis and occasionally to a complicated course. We present one of such atypical initial presentations which later on had a complicated course.

## Case presentation

An 11-year-old boy was referred to the Dermatology outpatient clinic with a complaint of patchy hair loss on his scalp noticed by parents one month before. On examination, he had 3 x 2 cm patch of alopecia on the occiput, without scarring. A diagnosis of alopecia areata was considered and treated with topical steroids. Four months later, he presented with intermittent fever, cough and weight loss that started from the past 45 days. 

**Figure 1:**
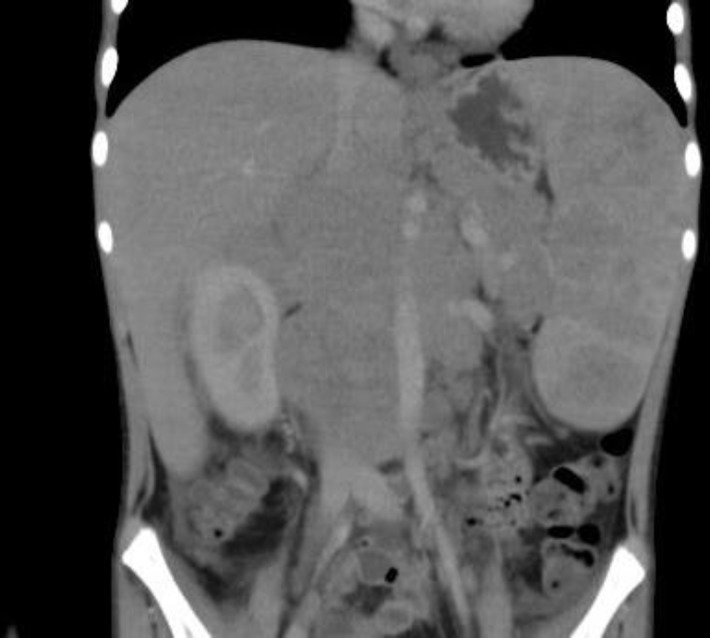
Contrast enhanced Computed Tomography (CECT) abdomen showing homogenously enhancing conglomerated mass forming lymph nodes in aortic and para-aortic region with encasement of abdominal aorta, IVC, and renal vessels extending from D11 to L4 vertebrae.

**Figure 2:**
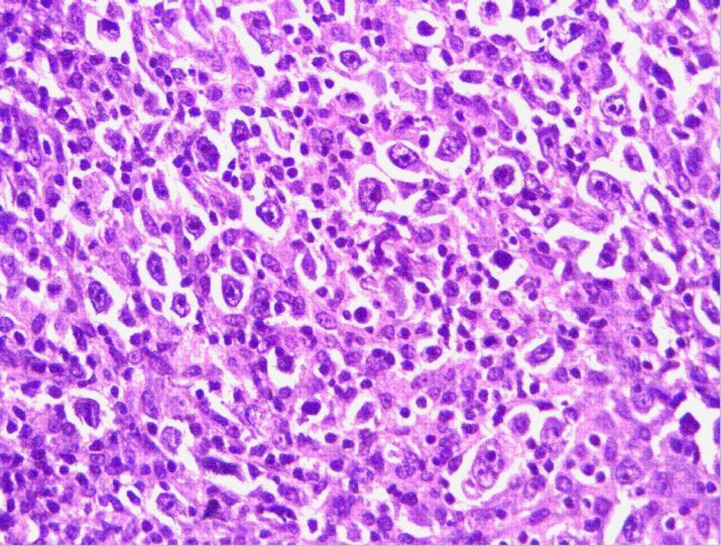
Biopsy of lymph node mass (Hematoxylin and eosin stain, magnification x 200) showing diffuse infiltration by mononuclear cells with large vesicular nuclei amnd occasional Reed Sternberg cells.

On examination, he was pale and cachexic with hepato-splenomegaly (16cm and 16.4cm, respectively). There was no cyanosis, clubbing, lymphadenopathy or pedal oedema. Alopecia over the scalp was regressing. Rest of the systemic examination was normal. Investigations showed anemia (Hb: 5.2 Gm%), elevated acute-phase reactants (ESR -99mm/hr, CRP-185mg/L) & Serum LDH levels (428IU/L) with hypo-albuminemia (3.14 Gm/dl). Mantoux test and serological tests for the diagnosis of HIV, HCV, HBsAg were negative. Sputum AFB and aerobic culture were also negative. USG abdomen and contrast-enhanced computed tomography (CECT) (Fig1) identified enlarged lymph nodes in the abdomen (extending from D11 to L4 vertebral level) and thorax (paratracheal), so a preliminary diagnosis of lymphoma was made. There was no evidence of consolidation or other abnormalities in CECT thorax. Bone marrow study showed no evidence of infiltration. A diagnostic laparoscopic biopsy was done, and histopathological (Fig-2) examination (Reed-Sternberg cells) and immunohistochemistry {positive CD15 & CD30 (membrane and Golgi zone), negative CD 20, CD 3, LCA, ALK 1} was consistent with classical Hodgkin's lymphoma (Lymphocyte-rich). A diagnosis of stage III B classical Hodgkin's lymphoma was considered.

**Figure 3:**
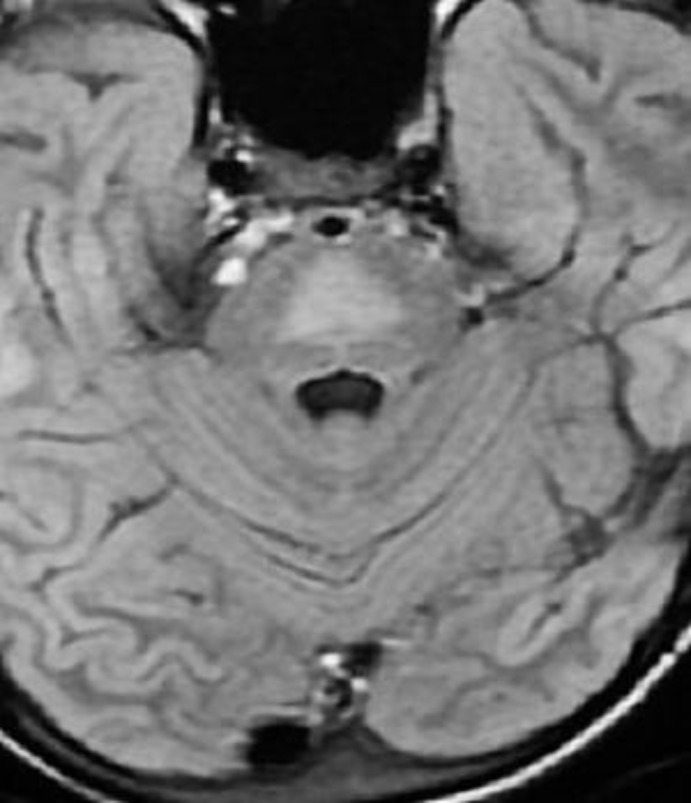
MRI brain, T2 Axial FLAIR showing trident shaped hyper-intensity in the central portion of pons.

**Figure 4:**
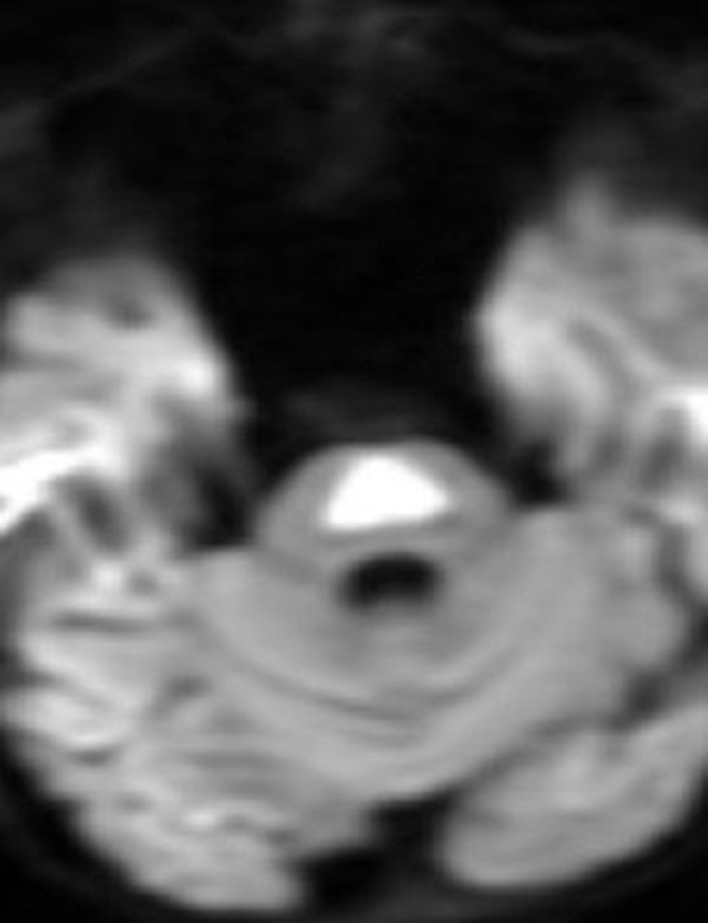
MRI brain, Axial DWI showing trident shaped diffusion restriction in the central portion of pons corresponding with fig-3

On the sixth post laparoscopic day, two episodes of generalised tonic-clonic seizures followed by altered sensorium were observed. Magnetic resonance imaging (MRI) brain (Fig-3, Fig-4) was suggestive of pontine demyelination. MRI showed no evidence of tumor deposits in brain. He was afebrile and had no dyselectrolytemia [sodium: 138meq/L (135-145mmol/L), Potassium: 4.1mmol/L(3.3-4.6mmol/L), Chloride: 102mmol/L (98-106mmol/L), Calcium:8.6mg/dl (8.4-10.2mg/dl), Phosphorous:3.4mg/dl (2.9-5.4mg/dl), Mg:1.8meq/L(1.5-2.3mg/dl)]. There were no rapid shifts in the electrolyte levels during hospitalization. Other causes of pontine myelinolysis were ruled out (AST: 25U/L (10-40U/L), ALT: 40U/L (5-45U/L), Serum albumin: 3.3gm/dl (3.5-5.6gm/dl), Plasma Ammonia: 24 micromol/L (11-35 micromol/L), Random blood sugar: 90mg/dl (60-140mg/dl), Serum urea: 16 (7-18mg/dl), serum ceruloplasmin:25mg/dl (15-45mg/dl), Urine copper:40mcg/24hrs (0-70mcg/24hrs) and a normal ANA profile). 

He was started on vitamin supplementation on day 1 of admission due to the malnourished state and was continued parenterally during and after the operative procedure. Neurologist opinion was obtained and a probable diagnosis of demyelination as a paraneoplastic manifestation of Hodgkin's lymphoma was considered as other casuses were ruled out. Cerebro-spinal fluid (CSF) analysis, including cell cytology to look for abnormal cells, was normal, and he was started on high-dose Methylprednisolone. Later, he developed a high-grade fever with seizures. Repeat CSF analysis revealed normal cell count and protein, no abnormal cells, but showed positive titers for HSV-2 (ELISA). Deterioration of the clinical condition was attributed to HSV infection. Electroencephalogram (EEG) showed a severe diffuse disturbance of electrical function, so he was started on IV Acyclovir and continued on antiepileptics (Levetiracetam 50mg/kg/day & Phenobarbitone 6mg/kg/day). Seizures were controlled; however, he sustained neurological deficits (spasticity& dystonia). He was started on chemotherapy (ABVD) once his general condition improved. Abdominal lymph nodes and hepatosplenomegaly regressed following chemotherapy and his functional capacity for basic work activities (i.e., the ability to sit, walk with support and feed himself) improved over a period of time.

## Discussion

 Lymphadenopathy is the most common initial presentation of Hodgkin’s lymphoma with head and neck most commonly involved followed by thorax and abdomen. The common sites for the extranodal disease include bone marrow, lung, liver, pericardium and pleura. Few patients may present with B symptoms (fever, drenching night sweats and unexplained weight loss of greater than or equal to 10% of body weight). Diagnosis is aided by biopsy and Immunohistochemistry ^2^^,^^3^ .

The World Health Organization (WHO) classified Hodgkin’s lymphoma into broadly two categories: the nodular lymphocyte predominant Hodgkin’s lymphoma (NLPHL) comprising of 5% of all HL and classical HL. Classical HL is further divided into four subtypes: nodular sclerosis (NS), mixed cellularity (MC), lymphocyte-rich (LR) and lymphocyte depleted (LD) comprising about 70%, 13-20%, 3-5% and 1-5% of all HL cases, respectively ^3^^-^^5^^.^

HL is highly responsive to chemotherapy and radiotherapy with event-free survival at five years ranging from 63-90% depending on the stage of disease and the regimen used. Secondary malignancies, endocrine disorders, cardiomyopathy and infertility are the significant side-effects of chemotherapy^6^.

Alopecia areata (AA) is associated with auto-immune disorders (thyroid disorders, vitiligo, pernicious anemia, diabetes, lupus erythematosus, myasthenia gravis, rheumatoid arthritis, and ulcerative colitis), Hodgkin’s lymphoma, non-Hodgkin’s lymphoma and multiple myeloma^3^^,^^7^. Antibodies against pigmented hair follicles, impaired T-cell function with increased T-helper/T-suppressor cell ratio, loss of the immune privilege of proximal anagen hair follicle and aberrant expression of cytokines are the possible underlying mechanisms in AA^7^. In Hodgkin’s lymphoma, alopecia areata is most often a paraneoplastic manifestation and in few cases it is related to direct infiltration of skin with malignant cells^8^^-^^10^. In a study by Anderson et al., alopecia areata was associated with <0.004% of Hodgkin's lymphoma cases^11^. Mlczoch et al. described alopecia areata in a 17-year-old patient with stage IV Hodgkin's lymphoma^12^. Alopecia areata can either precede or occur concurrently along with systemic manifestations^13^. In the present case, alopecia preceded systemic symptoms by approximately 4 months, highlighting the importance of maintaining a high index of suspicion in such cases where early diagnosis can improve the outcome as well as prevent significant morbidity due to complications of the primary disease.

Paraneoplastic neurological syndromes are rarely associated with cancer. PNS are associated with antibodies directed against antigens expressed by the tumor, suggest that these disorders are immune-mediated ^14^. In PNS, antibodies are triggered by the immune system against the antigens that are normally present in the nervous system and are ectopically expressed by the tumor, thereby causing varied clinical manifestations depending on the part of CNS involved ^15^. In less than 1% of cases, Hodgkin’s lymphomas are associated with PNS and most often include posterior cerebellar degeneration, limbic encephalitis, granulomatous angitis of central nervous system, dermatomyositis and sensorimotor neuropathies^14^^,^^16^. In the present case, the patient had pontine myelinolysis which is a non-classical neurologic syndrome, and due to the concurrent presence of tumor and improvement following chemotherapy, it can be considered as a definite paraneoplastic neurological syndrome. The utilization of onconeural antibodies in definitive diagnosis is an evolving research but, at present, it has its limitations with sensitivity, specificity, availability and standardization of the methodology^14^. PNS may occur without onconeural antibodies, and the antibodies can occur without a neurological syndrome^14^^,^^15^.

## CONCLUSION

 Varied presentation of malignancies necessitates a sound knowledge about associated paraneoplastic syndromes. A high index of suspicion in alopecia areata and detailed search for the primary site will clinch the diagnosis at earliest, avert many complications and improve the quality of life among patients.
